# Classification of Honey Powder Composition by FTIR Spectroscopy Coupled with Chemometric Analysis

**DOI:** 10.3390/molecules27123800

**Published:** 2022-06-13

**Authors:** Arkadiusz Matwijczuk, Iwona Budziak-Wieczorek, Grzegorz Czernel, Dariusz Karcz, Alicja Barańska, Aleksandra Jedlińska, Katarzyna Samborska

**Affiliations:** 1 Department of Biophysics, Faculty of Environmental Biology, University of Life Sciences in Lublin, Akademicka 13, 20-950 Lublin, Poland; grzegorz.czernel@up.lublin.pl; 2 Department of Chemistry, Faculty of Life Science and Biotechnology, University of Life Sciences in Lublin, Akademicka 15, 20-950 Lublin, Poland; iwona.budziak@up.lublin.pl; 3 Department of Chemical Technology and Environmental Analytics (C1), Faculty of Chemical Engineering and Technology, Cracow University of Technology, Warszawska 24, 31-155 Kraków, Poland; dariusz.karcz@pk.edu.pl; 4 Department of Food Engineering and Process Management, Institute of Food Sciences, Warsaw University of Life Sciences WULS-SGGW, Nowoursynowska 159c, 02-776 Warsaw, Poland; alicja_baranska@sggw.edu.pl (A.B.); aleksandra_jedlinska@sggw.edu.pl (A.J.)

**Keywords:** honey powder, spray drying, carrier, FTIR spectroscopy, chemometric analysis

## Abstract

Fourier transform infrared spectroscopy (FTIR) in connection with chemometric analysis were used as a fast and direct approach to classify spray dried honey powder compositions in terms of honey content, the type of diluent (water or skim milk), and carrier (maltodextrin or skim milk powder) used for the preparation of feed solutions before spray drying. Eleven variants of honey powders containing different amounts of honey, the type of carrier, and the diluent were investigated and compared to pure honey and carrier materials. Chemometric discrimination of samples was achieved by principal component analysis (PCA), hierarchical clustering analysis (HCA), linear discriminant analysis (LDA), and partial least squares-discriminant analysis (PLS-DA) modelling procedures performed on the FTIR preprocessed spectral data for the fingerprint region (1800–750 cm^−1^) and the extended region (3600–750 cm^−1^). As a result, it was noticed that the type of carrier is a significant factor during the classification of different samples of powdered multifloral honey. PCA divided the samples based on the type of carrier, and additionally among maltodextrin-honey powders it was possible to distinguish the type of diluent. The result obtained by PCA-LDA and PLS-DA scores yielded a clear separation between four classes of samples and showed a very good discrimination between the different honey powder with a 100.0% correct overall classification rate of the samples.

## 1. Introduction

The production of honey in the powdered form is the method to preserve this product, which is vulnerable for crystallization. Crystallization affects the stability of honey, because free water that is released during this process is a good environment for yeast growth and fermentation. Moreover, honey in the powder form can be more easily used as the component of various foods and dietary supplements [1]. Due to the formulated flowable form, the handling, dosage, and transportation of honey powder is easier and cheaper than in a liquid or crystallized form. However, the dehydration of honey requires the application of additional substances—drying carriers—which increase the glass transition temperature (*T_g_*) and makes the process possible. Without a carrier, as a consequence of honey’s low *T_g_*, it is not possible to obtain the powdered form, because even at low water content honey stays in the syrup-like form [2,3]. Among the carriers, the most popular substances are maltodextrins and gum Arabic. However, the application of such substances is becoming worse perceived by consumers. That is why new types of carriers are still needed for the preparation of honey powder. Such a new carrier can be milk powder. Due to the presence of lactose, the *T_g_* of milk powder is high enough to enhance the drying process. Additionally, the presence of milk proteins also enhances the process, because proteins create a shell on the external layer of droplets during atomization, and it reduces the stickiness [4]. Due to the fact that the number of carrier types applied for honey powder production increases, there is a need for fast, cheap, and reliable evaluation and differentiation methods.

In this study FTIR was applied in order to estimate the molecular differences in spectroscopic spectra for multifloral honey samples powdered with the different carriers and diluents. This research method is currently gaining popularity in the context of analysing food products with respect to their potential health benefits, e.g., honeys, oils, juices, etc. The largest chances between powdered honey and honey products in the FTIR spectra occurred in the range of 3600–3000 cm^−1^, and correspond to the stretching vibration of the –OH group of carbohydrates, water, and organic acids. Changes in the spectral region corresponding to vibrations of the anomeric region of carbohydrates or C–H and C–C deformation (900–700 cm^−1^) provides good information about the changes in samples. Its value results from its speed, non-invasiveness, and, above all, reliability of the results. Using FTIR spectroscopy with exploratory multivariate analysis such as principal component analysis (PCA), hierarchical cluster analysis (HCA), linear discriminant analysis (LDA), and partial least squares-discriminant analysis (PLS-DA) is a rapid method during the identification of food samples. PCA and HCA belong to unsupervised methods and can be use simultaneously to analyse the data structure and find similarities between multiple samples. LDA and PLS-DA are supervised classification methods that can be used for predictive and descriptive modelling for a high-dimensional spectroscopic dataset. In the literature there is a lot of examples of the application of FTIR spectroscopy in a combination with chemometric techniques to differentiate honey from different botanical origins and study the authentication and quality [5,6,7,8,9,10], but none of them are devoted to honey powders obtained by spray drying with different carriers. 

## 2. Materials and Methods

### 2.1. Materials 

Multifloral honey (H) was purchased from a local apiarist (Siemiatycze, Poland). Maltodextrin (MD), characterized by DE 17.0-19.9, from Tate and Lyle (Boleráz, Slovakia), and skim milk powder (MP) from Mlekovita (Warsaw, Poland) were used as carrier materials. Skimmed milk (m) from Mlekovita (Warsaw, Poland) was used as water substitution for feed solution preparation in selected variants. 

### 2.2. Feed Solutions Preparation and Spray Drying 

Twelve variants of honey/carrier solutions were prepared by mechanical mixing with water or skimmed milk (Table 1). Honey to carrier solids ratio varied, it was 40:60, 50:50, 60:40, an 70:30. Higher amount of honey addition was possible when MP was used as a carrier due to its positive influence on stickiness reduction during spray drying. Thus, variants were allocated into five groups: I—pure components (H, MD, and MP), II—honey with maltodextrin dissolved in water, III—honey with milk powder dissolved in water, IV—honey with maltodextrin dissolved in skim milk, and V—honey with milk powder dissolved in skim milk.

NIRO MINOR laboratory spray drier (GEA, Skanderborg, Denmark) was applied at the following conditions: feed rate 0.22 mL∙s^−1^, rotary speed of atomizing disc 26,000 rpm (compressed air pressure 4.5 bar), inlet/outlet air temperature 180/80 °C. Spray drying procedure was performed in duplicate for each experimental variant. Variant 60MDw was not successfully transformed into powder due to high stickiness, so for chemometric analysis 11 honey powders were directed, as well as 3 basic materials for a comparison: honey, maltodextrin, and skim milk powder.

### 2.3. ATR-FTIR Measurement 

Measurements of infrared spectra for 14 analysed samples (11 honey powders and 3 basic materials: honey, maltodextrin, and milk powder) were conducted with the use of a 670-IR spectrometer (Agilent, Santa Clara, CA, USA) at 23 °C. An ATR (Attenuated Total Reflection) attachment was used in the form of a ZnSe crystal with adequate geometry (truncated at 45°) to ensure 20-fold internal reflection of the absorbed beam. The powders were pressed onto the crystal surface. During the measurement, 16 scans were registered and subsequently, the programme averaged the results for all spectra. Prior to the measurement, the ZnSe crystal was cleaned using ultra-pure organic solvents by Sigma-Aldrich (Darmstadt, Germany). Prior to (1h) and during the experiment, the measurement chamber was kept in an inert N_2_ atmosphere. Spectral measurements were recorded in the region from 500 to 4000 cm^−1^ at the resolution of 1 cm^−1^. 

### 2.4. Chemometrics Analysis 

Spectral pre-processing was processed with the use of Grams/AI 8.0 software (Thermo Scientific, Waltham, MA, USA) and OriginPro (OriginLab Corporation, Northampton, MA, USA). Before the chemometric analysis the spectra were subjected to pre-treatment using multi-point baseline correction, Y offset correlation, mean center, and second-order derivative (Savitzky-Golay smoothing algorithm with 20 points).

Multivariate analysis such as principal component analysis (PCA), hierarchical cluster analysis (HCA), linear discriminant analysis (LDA), and partial least squares-discriminant analysis (PLS-DA) were performed for the FTIR spectra. Chemometrics analysis were conducted in the broad range of spectra 3600–750 cm^−1^ and in the fingerprint region 1800–750 cm^−1^. The chemometrics analysis were performed using the Statistica 13 software (TIBCO Software Inc. Palo Alto, CA, USA) and XLSTAT (Addinsoft Inc., New York, NY, USA).

#### 2.4.1. Principal Component Analysis (PCA)

Principal component analysis (PCA) is a mathematical procedure used for the reduction of dimension and compression datasets into a lesser number of uncorrelated variables called principal components (PC) [11,12]. PCs are orthogonal to each other and usually the first component explains the largest possible variances. The goal of PCA is to obtain multiple-variable system to detect data structure, enable the classification between samples, and determine general relationship among data [13]. The PCA is an exploratory technique, which is based on the following expression:

(1) $$X={\mathrm{TP}}^{T}+E$$

where X is the data matrix to be analysed, T is called score matrix, P the loading matrix, and E the residual. The non-linear iterative partial least squares (NIPALS) algorithm is the most commonly used method for constructing PCA models (determine principal components) [14].

The non-linear iterative partial least squares (NIPALS) algorithm was used to perform PCA analysis. The principal components, which respond to the information resources about the studied phenomenon, were determined so that the variances of the next principal components were getting smaller. The selection of the appropriate number of principal components included in the analysis can be facilitated by analysis of percentage of variances. Cumulative sum of variances included in the principal components analysis should exceed a certain degree, usually it is about 80% [15]. Each component in terms of the variables can be characterized by evaluating the loadings. The loading plot is the correlation coefficients between the variables and factors and can range from −1 to 1. Loadings close to −1 or 1 indicate that the variable is strongly correlated to component [16].

#### 2.4.2. Hierarchical Clustering Analysis (HCA)

Hierarchical clustering analysis (HCA) is one of the exploratory methods used to classify items into clusters (group) based on distance that is calculated from distance matrix and the similarity between them [5,17]. Graphical representation of results is a tree graph called a dendrogram. HCA is based on finding the smallest distances between items (such as spectroscopic spectra) and the measure of dissimilarity between sets of observations. In hierarchical clustering the appropriate method of measuring the distance between pairs of observations and linkage criteria should be used. As it is distinct from other clustering algorithms, in this case it is not necessary to predeterminate the number of created clusters [18]. 

In HCA, Pearson correlation and Manhattan distance between the pairs of samples were used as a distance measure and the average linkage and complete linkage criteria were used as an agglomeration method. Graphical representation of results is a tree graph called a dendrogram.

#### 2.4.3. Linear Discriminant Analysis (LDA)

Linear Discriminant Analysis (LDA) is a dimensional reduction technique that is used for supervised classification problems [19,20]. LDA is used to classify objects (such as spectroscopic spectra) by a discriminant function from linear combinations of variables that give maximum differentiation between groups. It can be achieved by means of a mathematical classification algorithm based on a Mahalanobis distance calculation [21]. Establishing a classification model enables the prediction of an unknown sample to the most probable class. LDA method cannot be applied for high dimensionality of the data when the number of spectral variables is larger than the number of samples. For this reason, LDA can be applied to the PCA scores based on the first extracted PCs.

LDA was conducted in the 1800–750 cm^−1^ spectral region based on three first PCs from PCA.

#### 2.4.4. Partial Least Squares-Discriminant Analysis (PLS-DA)

Partial least squares discriminant analysis (PLS-DA) is one of the most widely used supervised classification technique in chemometrics that combines partial least squares regression (PLS) with LDA [22]. PLS-DA model is used to optimize separation between different groups of samples and is completed by linking two matrices: X (the spectral data) and dependent variable (groups, class membership etc.). PLS constructs a linear regression model by projecting the predictor variables and response variables to a new set of latent variables (LVs), called factors, with maximum covariance [23]

PLS-DA was performed in the fingerprint region 1800–750 cm^−1^. PLS-DA models were extracted at a confidence level of 95%

## 3. Results and Discussion

### 3.1. FTIR

**Figure 1:**
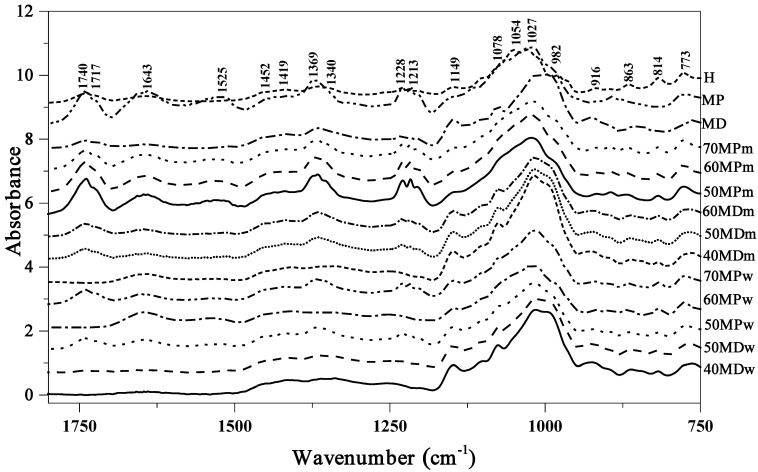
FTIR spectra for different samples of honey and basic materials. All spectra were normalized at the 3290 cm^−1^.

The samples were analysed using ATR-FTIR spectroscopy. In order to facilitate an easier presentation of the bands and their characteristics in Figure 1, Table 2, and Table S1 (within the spectral range of 3330–800 cm^−1^), the spectra for the respective samples were represented and correlated with the matching functional group vibrations. FTIR spectra for pure components (maltodextrin, milk powder, and honey) are presented additionally in Supplementary Materials in Figure S1. As observed in [7,8], the first clearly visible region of vibrations in the analysed samples is found within the range of 3650 to approx. 3000 cm^−1^ (for all samples Figure 1). The region is characteristic for the stretching vibrations of –OH groups in carbohydrates, water, and organic acids. For this type of sample, it is not uncommon to see also the stretching vibrations of carboxylic acids as well as the NH_3_ stretching band of free amino acids in this region. Next, in the region of 3000–2800 cm^−1^, we observed the stretching vibrations of C–H groups (both alkaline and aromatic, as present in the chemical sugar skeleton). Vibrations with the maximum at ~3290 cm^−1^ belong to the characteristic contribution of carboxylic acids, whose irregular absorption (with a wide –OH band) enhances the stretching vibrations of C–H groups. The broad bands of ν(–OH) vibrations result from the formation of strong hydrogen bonds belonging to carboxylic acid dimers [7]. The deformation vibrations of –OH groups also correspond to the band with the maximum at ~1643 cm^−1^ (Figure 1).

A very important region only weakly present in our spectra, with the maximum at approx. 1717 cm^−1^, is due to the stretching vibrations of functional groups in ketones, C=O in fructose, and aldehyde CH=O in glucose (in practice, it enhances only the band with the max. at 1643 cm^−1^). The very characteristic fingerprint region of the selected samples covers the range of 1480 to 700 cm^−1^ and includes a variety of bands. The most important include: the stretching vibrations of C–O, C–C, and C–H, as well as the bending vibrations of C–H present in the chemical structure of carbohydrates [7,24] (very often originating also from organic acids and carotenes). For the milk powder, two characteristic regions were present: 1541–1547 cm^−1^, which is characteristic for the bending vibration N–H in amide II, and 1241–1251 cm^−1^, which establishes the stretching mode C–N in amide III [23,25]. The most interesting vibrations in this region are: the bands at 1452 cm^−1^, 1419 cm^−1^, and 1340 to 1264 cm^−1^. They originate primarily from the deformation vibrations of O-CH and C–C–H groups in the carbohydrate structure, and deformation vibrations belonging to the δ-OH groups in the C–OH structure. The very important bands within the range from 1245 to 958 cm^−1^ are the stretching vibrations of C–H groups or (C–O) in carbohydrate structures. The bands at 1145 and 1027 cm^−1^ originate from the vibrations of C–O groups in C–O–C. The region from 1027 to 958 cm^−1^ as well as below 900 cm^−1^ contain the stretching vibrations of C–O in the C–OH group or C–C stretching in the carbohydrate structure [8,26,27]. The last of the presented regions: 900–700 cm^−1^ is characteristic for the vibrations of the anomeric region of carbohydrates or C–H and C–C deformation [27,28]. Changes in this spectral region often evidence relatively strong modifications of the sugar fraction bonds. 

### 3.2. Hierarchical Clustering Analysis (HCA)

Hierarchical clustering analysis was used to visualize the base classification of the group and sub-group arrangement of powdered multifloral honey from FTIR spectra. The HCA focus mainly on finding similarities between samples by the use of different classification algorithms. In this analysis two spectral regions were taken into account. For the fingerprint region Euclidean distance, complete linkage criterion, and average linkage criterion were used, and for the 3600-750 cm^−1^ region Euclidean distance and average linkage criterion were applied. Our main goal of the multivariate analysis was to compare the powdered multifloral honey (11 samples). Additionally, we performed HCA for all 14 samples (basic materials and powdered multifloral honey), as a comparative analysis. 

**Figure 2:**
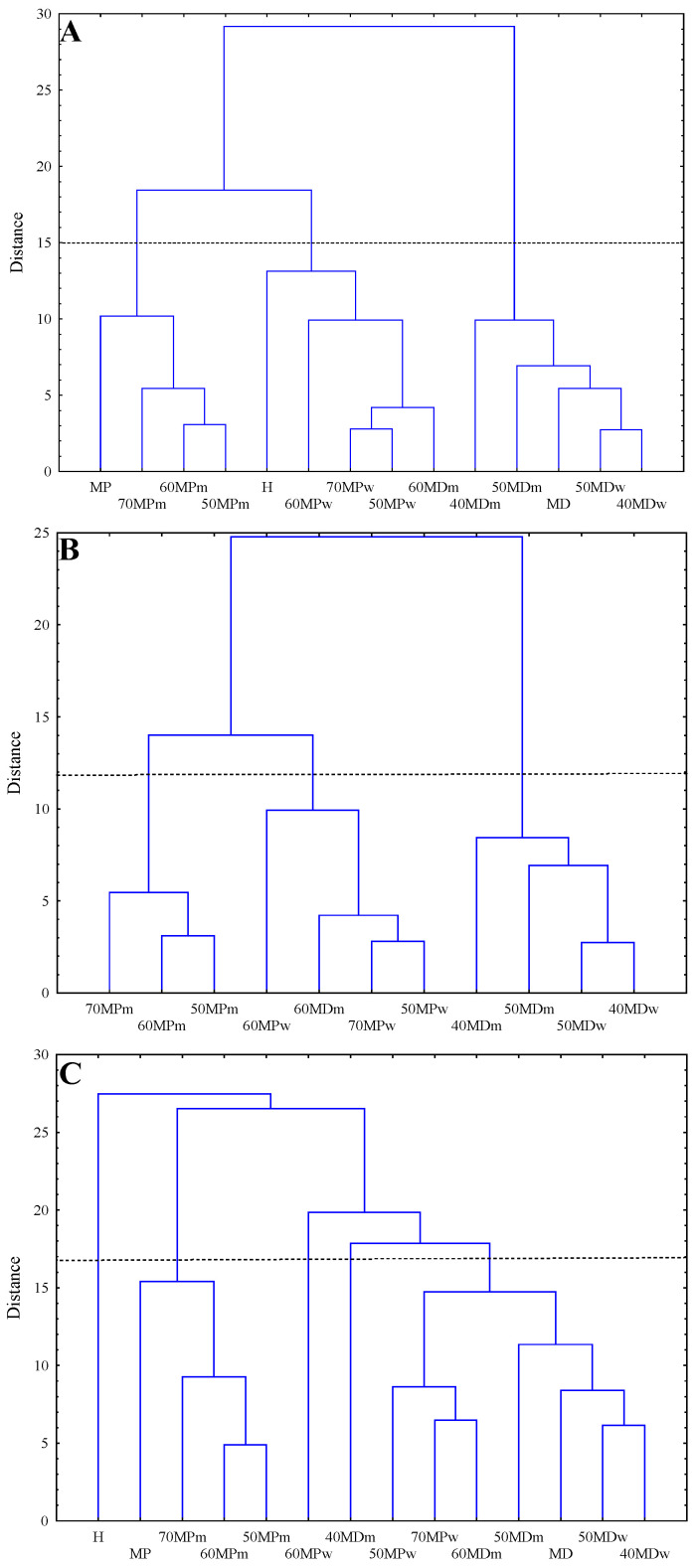
Hierarchical clustering analysis (HCA) tree diagram. (**A**)—analysis performed on all 14 samples in the range between 1800–750 cm^−1^, (**B**)—analysis performed on 11 samples from II–V group in the range between 1800–750 cm^−1^, and (**C**)—analysis performed on all 14 samples in the range between 3600–750 cm^−1^.

In Figure 2 tree diagrams are presented. In Figure 2A,B (the spectral range of 750–1800 cm^−1^) the resulting dendrogram grouped the samples into three major clusters. Honey powder samples that contain skim milk powder as a carrier clearly split into two groups, while samples containing maltodextrin as a carrier with different diluents revealed similarity. In panel A it can be observed that the MP sample shows similarity to MPm samples, the H sample shows similarity to the MPw samples, and the MD sample shows similarity to the MDm and MDw samples. For the second broader region (Figure 2C), slightly similar results were obtained as from the fingerprint region. In this case, one notable cluster was formed from samples 50MPm, 60MPm, 70MPm, and MP, meanwhile the second cluster was more elaborate. The pure material of honey (H) was clearly separated from other samples. 

Although the results obtained from the HCA revealed differences of honey powders based on the different type of carrier and diluent, other multivariate methods such as PCA-LDA and PLS-DA must be used in order to construct the confident models with the ability to predict the class membership of the powdered honey.

### 3.3. Principal Components Analysis (PCA) and Linear Discriminant Analysis (LDA)

In this study, the discrimination of the powdered honey based on FTIR spectra was performed by a combination of PCA, followed by an LDA. PCA was applied to estimate the systematic variation in a data matrix by a low-dimensional model plane, which allowed a better visualization of the data and describe a complex data set by a few numbers of PCs. LDA was performed on the first three PCA scores in order to calculate discriminant functions for the classification on honey samples.

PCA were conducted on multifloral honey samples based on FTIR spectra over the range of the fingerprint region 1800–750 cm^−1^ (11 and 14 samples such as in HCA) as well as 750–3600 cm^−1^ for better comparison. The contribution of total variance and eigenvalues for the selected regions are presented in Table 3. Better differentiation was achieved for the fingerprint region where the first three principal components explained almost 90% (14 samples) and 97% (11 samples) of total variance while for the broad range only 84%. Therefore, further analysis was performed for the fingerprint region.

**Figure 3:**
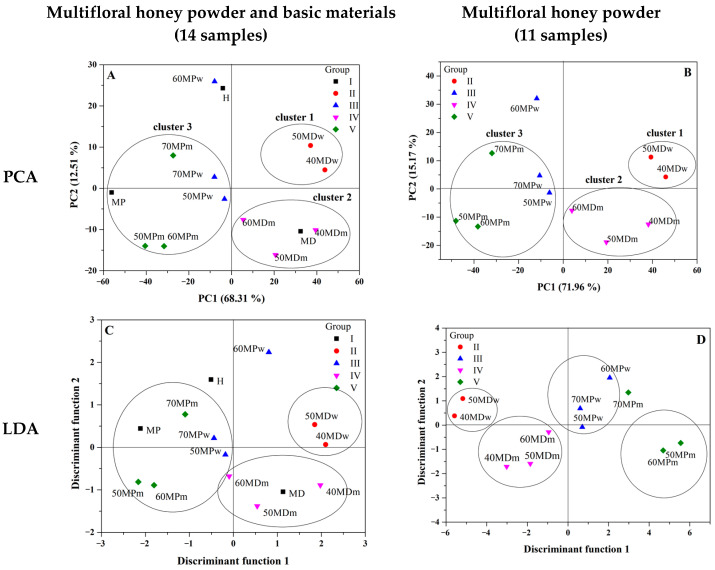
Principal component analysis (PCA) two-dimensional (2D) score plot (PC1 versus PC2) calculated for the data acquired from the FTIR spectra in the range between 1800—750 cm^−1^. (**A**)—analysis conducted for 14 samples (I-V group) and (**B**)—analysis conducted for 11 samples (II-V group). Linear Discriminant Analysis (LDA) score plot of multifloral honeys of different carrier and diluent by using first three PCs as variables. (**C**)—analysis conducted for 14 samples (I-V group) and (**D**)—analysis conducted for 11 samples (II-V group). Range between 1800–750 cm^−1^.

Figure 3A,B shows a score plot in two-dimensional projection for all 14 samples and 11 powdered multifloral honey samples. In Figure 3A,B the creation of three clusters can be observed: the first cluster consisted of samples of honeys 40MDw and 50MDw, with maltodextrin, dissolved in water, which were positively correlated with PC1 and PC2. The second cluster consisted of 40MDm, 50MDm, and 60MDmm, honeys with maltodextrin, dissolved in skim milk. The third cluster contained MPw and MPm honey samples with skim milk powder dissolved in skim milk or water. The main result obtained from the PCA is that the type of carrier used has a significant impact for sub-group arrangement and the classification into clusters. Honey samples with skim milk powder dissolved in water or milk showed similarity and formed a separate cluster three (negative correlation to PC1). Moreover, honey samples with maltodextrin, depending on the type of diluent, were located in different clusters at the score plot graph. Thus, it was possible to distinguish the type of diluent in such types of honey powder. It can be said that the type of carrier and diluent are much more important factors during sub-group arrangement in comparison to the honey solid to carrier solid ratio content.

**Figure 4:**
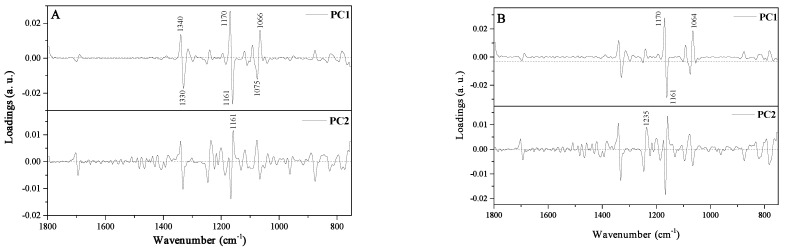
The loading plot of PC1 (on the top) and PC2 (on the bottom) for the region 750–1800 cm^−1^. (**A**)—analysis conducted for 14 samples (I-V group) and (**B**)—analysis conducted for 11 samples (II–V group).

The loading plot indicated which variables possess the greatest influence on score as well as which have the highest and the least contribution to creating the principal component [29]. The PCA loading plot in Figure 4A,B reveals that PC1 was positively correlated with the bands at 1066, 1170, and 1340 cm^−1^ assigned to stretching vibrations on C-O in the C-O-C and C-H groups in the carbohydrate structure. The fingerprint region has significant influence into the classification between different samples of honey due to a unique spectrum specific for every polysaccharide [5].

Then, LDA was performed by using the first three principal components as variables. Figure 3C (all 14 samples) and 3D (11 samples) show the LDA score plot representing the first two discriminant functions. According to the Figure 3D a good degree of discriminant among powdered multifloral honey samples was achieved when basic components (MD, MP, and H) were not added to the analysis. According to Figure 3D, powdered multifloral samples were completely separated into four groups (II–V). The analysis showed a clear difference between the used carrier and diluent. Samples with skim milk powder as a carrier grouped on the positive side of the first linear discriminant function (LD1), while samples with maltodextrin grouped on the negative side of LD1. Additionally, samples with water as a diluent grouped on the positive side of LD2, while samples with skim milk grouped on the negative side of LD2. The discriminant model for all 14 samples allowed the correct classification of all samples into their respective groups (I–V) with a success rate of 57.1%. However, the model for 11 samples allows us to discriminate the groups with a 100% correct classification rate without error (Table 4).

### 3.4. The Partial Least Squares-Discriminant Analysis (PLS-DA)

PLS-DA supervised classification methods were employed to obtain a better discrimination of the FTIR spectra of powdered multifloral honey. For a total of 11 samples of multifloral honey powder (five samples contain maltodextrin carrier and six samples contain skim milk powder carrier), the model used the spectral range 1800–750 cm^−1^. The resulting dataset obtained by cross-validation using four latent variables (LV) was then randomly divided into training (nine samples) and test (two samples: 50MPw and 50MPm) set. 

**Figure 5:**
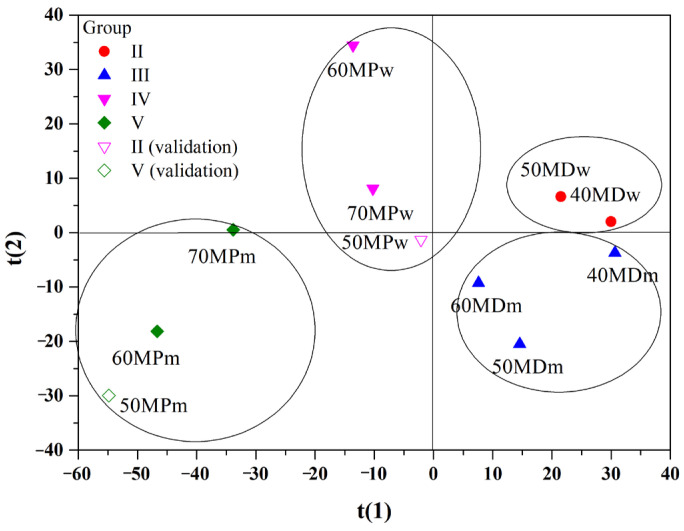
Partial least-squares-discriminant analysis (PLS-DA) scores plot t(1)/t(2) of FTIR spectra on the range 1800–750 cm^−1^.

The PLS–DA, carried out on multifloral honey powder also revealed in this case a clear separation among the sample classes. Thus, the resulting model was characterized with good statistical parameters (four components gave R^2^X = 0.889; R^2^Y = 0.907, Q^2^ = 0.607). The score plot for the first two latent variables is presented in Figure 5. By visual analysis of t(1)/t(2) scores-plot (Figure 5), the samples with maltodextrin (MDw and MDm) could be observed as two closely related groups at positive values of component t(1), while samples with skim milk powder (MPw and MPm) created two clearly separated groups at negative values of component t(1). In this case, the type of diluent was also important. Samples with water as a diluent were on the positive side of component t(2) and samples with skim milk were on the opposite side (negative t(2)).

This result was confirmed by confusion matrix (Table 5), which carries information about the predicted and actual classifications of samples. Table 5 summarises the correct classification rates for powdered multifloral honey after PLS-DA for training and validation samples. For the training set, PLS-DA discriminates the groups with a 100% correct classification without error and for the validation set a 50% correct classification.

## 4. Conclusions

FTIR coupled with modern multivariate analysis responding to the current needs for economic, simple, and fast methods able to classify samples with great accuracy were applied for honey powder classifications. The samples differed in the type of carrier and diluent, as well as the honey content. The analysis of FTIR spectra of honey samples with distinct types of carriers (maltodextrin and milk powder) revealed significant differences between them. Chemometric analyses of PCA and HCA were shown to be useful for samples classification, under the condition that the fingerprint region of spectra was analyzed. However, the sensitivity of both methods was different. PCA let to distinguishing the type of carrier, and among maltodextrin-based samples it was also possible to separate the type of diluent. The amount of honey was the factor that was not detectable by the applied method. Supervised classification methods PCA-LDA and PLS-DA proved to be a significant chemometric tool to classify the honey powder sample according to the used carrier and diluent. The best discriminant quality was obtained when the basic materials (H, MD, and MP) were not used in the model. The result obtained by PCA-LDA and PLS-DA scores equaled a clear separation between four classes of samples: MDw, MDm, MPw, and MPm.

## Figures and Tables

**Table 1:** Composition of honey powders obtained with different carriers (MD—maltodextrin, —milk powder) and diluents (w—water, m—skim milk).

Variant	Group	Honey Solids to Carrier Solids Ratio (*w*/*w*)
40MDw	II	40:60
50MDw		50:50
60MDw		60:40 *
50MPw	III	50:50
60MPw		60:40
70MPw		70:30
40MDm	IV	40:60
50MDm		50:50
60MDm		60:40
50MPm	V	50:50
60MPm		60:40
70MPm		70:30

* drying not possible due to high stickiness.

**Table 2:** The location of the maxima of absorption bands FTIR with arrangement of appropriate vibrations selected for maltodextrin (MD), skim milk powder (MP), and multifloral honey (H) made in terms of spectral 3900–700 cm^−1^.

Type and Origin of Vibrations	FTIR Position of Bands (cm^−1^)		
	MD	MP	H
ν(O–H) in H_2_O	3291	3285	3281
ν(C–H) or/and ν(NH_3_) of free amino acids δ (O–H) from H_2_O	2927 2892	2927 2872	2937 2886
ν(C–O) from carbohydrate	1742	1739	1740
δ (O–H) from H_2_O	1645	1643	1642
δ (N–H) from amid II		1546	
δ (O–CH) and δ (C–C–H)	1453	1449	1450
δ (O–H) in C–OH group + δ (C–H) in the alkenes	1417	1419	1420
δ (–OH) in C–OH group	1363	1366	1366
ν(C–H) in carbohydrates or/and ν(C–O) in carbohydrates	1257 1230	1254 1229	1257 1229
ν(C–H) in carbohydrates or/and ν(C–O) in carbohydrates	1148	1148	1148
ν(C–O) in C–O–C group	1104 1076	1114 1066	1101 1076
ν(C–O) in C–OH group or ν (C–C) in the carbohydrate structure	1013 992	1020 992	1027 980
δ (C–H)	925	915	916
Anomeric region of carbohydrates or δ (C–H)	896 862 846 819	883 875 852 821	895 864 817

**Table 3:** Eigenvalues, percentage of variance, and cumulative percentage in the data used for the PCA calculations obtained for the multifloral honey, maltodextrin, and skim milk powder.

Principal Component Number	Eigenvalue	Percentage of Variance (%)	Cumulative (%)
750–1800 cm^−1^ (14 samples)			
1	1004.849	68.310	68.310
2	184.067	12.513	80.823
3	133.050	9.044	89.868
4	92.275	6.272	96.141
5	32.647	2.219	98.360
750–1800 cm^−1^ (11 samples)			
1	1058.583	71.963	71.963
2	223.148	15.169	87.133
3	147.245	10.009	97.143
4	18.230	1.239	98.382
5	1058.583	71.963	71.963
750–3600 cm^−1^ (14 samples)			
1	1643.594	41.182	41.182
2	1311.975	32.873	74.055
3	396.217	9.927	83.983
4	205.171	5.140	89.124
5	141.846	3.554	92.678

**Table 4:** Classification results of the classes computed by LDA.

LDA prediction matrix for 14 samples						
True class	Assigned to class					% Correct classification
	I	II	III	IV	V	
I	1	0	0	1	1	33.3
II	0	2	0	0	0	100.0
III	2	0	1	0	0	33.3
IV	1	0	0	2	0	66.7
V	1	0	0	0	2	66.7
Total	5	2	1	3	3	57.1
LDA prediction matrix for 11 samples						
True class	Assigned to class				% Correct classification	
	II	III	IV	V		
II	2	0	0	0	100.0	
III	0	3	0	0	100.0	
IV	0	0	3	0	100.0	
V	0	0	0	3	100.0	
Total	2	3	3	3	100.0	

**Table 5:** Classification results of the classes computed by PLS-DA for training sample and validation sample.

Confusion matrix for the training sample (variable group):						
from\to	2	3	4	5	Total	% correct
2	2	0	0	0	2	100.00%
3	0	3	0	0	3	100.00%
4	0	0	2	0	2	100.00%
5	0	0	0	2	2	100.00%
Total	2	3	2	2	9	100.00%
Confusion matrix for the validation sample (variable group):						
from\to	2	3	4	5	Total	% correct
2	0	0	0	0	0	0.00%
3	0	0	0	0	0	0.00%
4	0	1	0	0	1	0.00%
5	0	0	0	1	1	100.00%
Total	0	1	0	1	2	50.00%

## Data Availability

Not applicable.

## Supplementary Materials

The following supporting information can be downloaded at: https://www.mdpi.com/article/10.3390/molecules27123800/s1, Figure S1: FTIR spectra for basic materials: honey, maltodextrin, skim milk powder, Figure S2: FTIR spectra for different sample of honey: (A)—with maltodextrin (MD), (B)—with milk powder (MP), Table S1: The location of the maxima of absorption bands FTIR with arrangement of appropriate vibration for selected for different sample of multifloral honey made in terms of spectral 3900−700 cm^−1^.
