# Supplementary material for: Classification of Honey Powder Composition by FTIR Spectroscopy Coupled with Chemometric Analysis
Source: Molecules. 2022 Jun 13;27(12):3800. doi: 10.3390/molecules27123800 (PMC9229643; doi:10.3390/molecules27123800)
Supplement: Supplementary file 1 [file molecules-27-03800-s001.zip › molecules-1734382-supplementary.pdf]

Supplementary materials

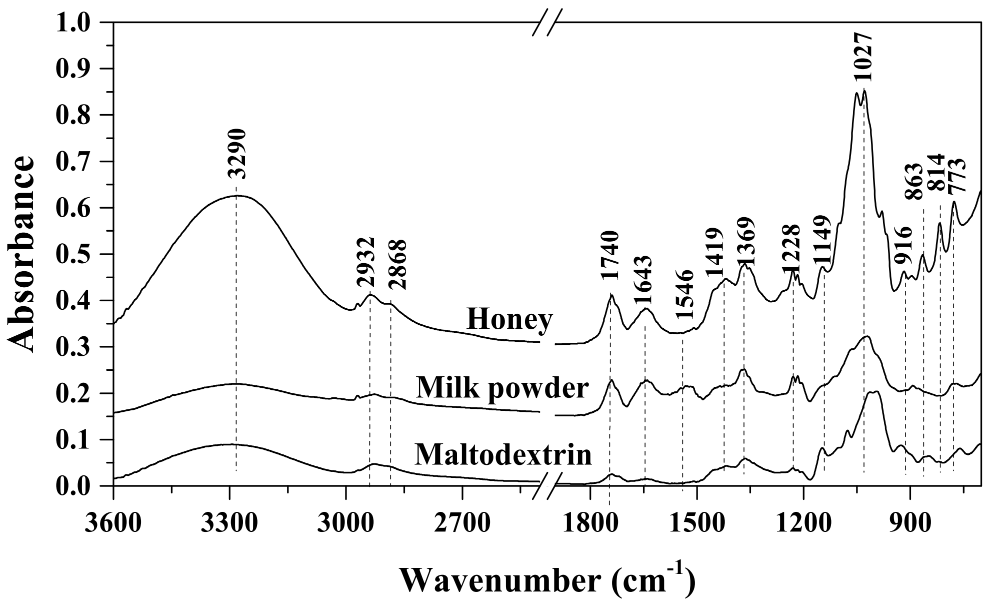

Figure S1. FTIR spectra for basic materials: honey, maltodextrin, skim milk powder.

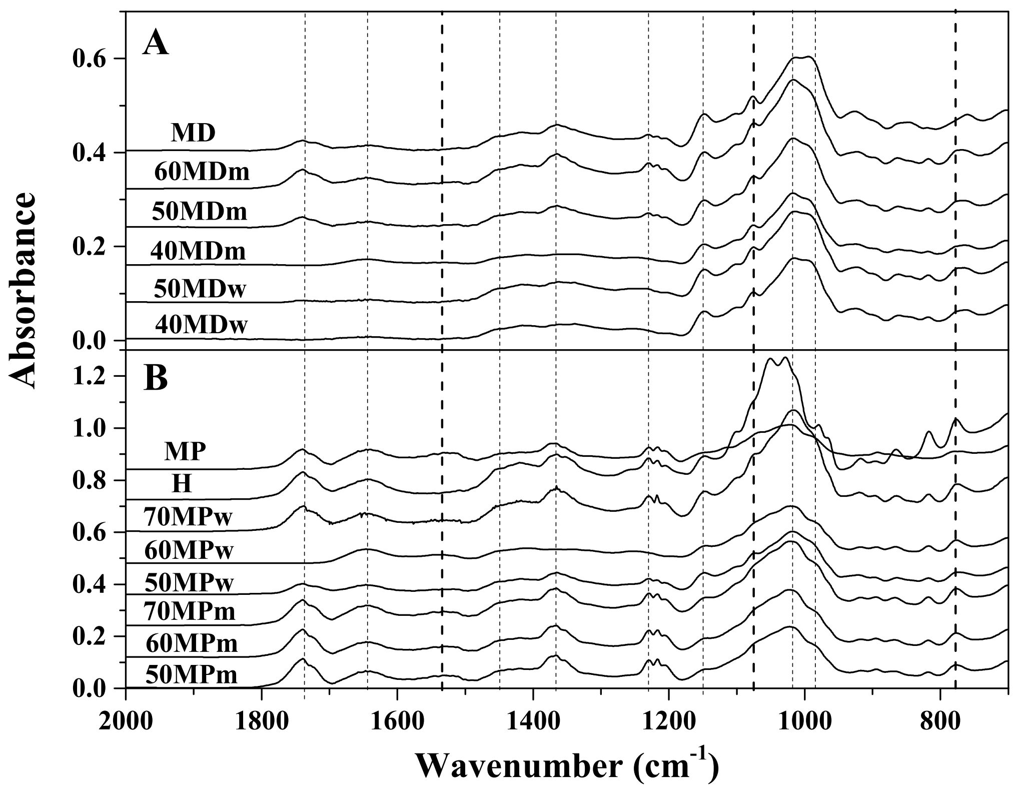

Figure S2. FTIR spectra for different sample of honey: (A) – with maltodextrin (MD), (B) – with milk powder (MP).

Table S1. The location of the maxima of absorption bands FTIR with arrangement of appropriate vibration for selected for different sample of multifloral honey made in terms of spectral 3900–700 cm^{-1}.

**Type and origin of vibrations**	**FTIR Position of bands (cm^{-1})**							
	**Basic materials - group I**			**Group II**		**Group III**		
	**MD**	**MP**	**H**	**40MDw**	**50MDw**	**40MDm**	**50MDm**	**60MDm**
ν (O–H) in H_2O	3291	3285	3281	3290	3291	3285	3286	3286
ν (C–H) or/and ν (NH_3) of free amino acids<br>δ (O–H) from H_2O	2927<br>2892	2927<br>2872	2937<br>2886	2924<br>2894	2927<br>2891	2922<br>2885	2926<br>2888	2927<br>2889
ν (C–O) from carbohydrate	1742	1739	1740	-	1740		1740	1740
δ (O–H) from H_2O	1645	1643	1642	1640	1647	1645	1645	1645
δ (N–H) from amid II		1546						
δ (O–CH) and δ (C–C–H)	1453	1449	1450	1453	1449	1446	1447	1450
δ (O–H) in C–OH group + δ (C–H) in the alkenes	1417	1419	1420	1413	1416	1410	1417	1417
δ (–OH) in C–OH group	1363	1366	1366	1361	1362	1360	1363	1363
ν (C–H) in carbohydrates or/and ν (C–O) in carbohydrates	1257<br>1229	1254<br>1229	1257<br>1229	1246<br>1198	1252<br>1235	1244	1257<br>1229	1257<br>1229
ν (C–H) in carbohydrates or/and ν	1148	1148	1148	1148	1148	1149	1148	1148

(C–O) in carbohydrates								
ν (C–O) in C–O–C group	1104<br>1077	1114<br>1066	1101<br>1076	1101<br>1077	1102<br>1077	1102<br>1077	1102<br>1077	1102<br>1077
ν (C–O) in C–OH group or ν (C–C) in the carbohydrate structure	1014<br>992	1017<br>992	1027<br>980	1015<br>993	1014<br>993	1016<br>994	1016<br>993	1016<br>992
δ (C–H)	925	915	916	924	924	923	922	922
anomeric region of carbohydrates or δ (C–H)	896<br>862<br>846<br>819	883<br>875<br>852<br>821	895<br>864<br>817	897<br>860<br>842<br>817	897<br>860<br>844<br>819	898<br>859<br>844<br>817	894<br>860<br>844<br>818	896<br>862<br>842<br>817

**Type and origin of vibrations**	**FTIR Position of bands (cm^{-1})**					
	**Group IV**			**Group V**		
	**50MPw**	**60MPw**	**70MPw**	**50MPm**	**60MPm**	**70MPm**
ν (O–H) in H_2O	3286	3286	3286	3281	3282	3286
ν (C–H) or/and ν (NH_3) of free amino acids δ (O–H) from H_2O	2928<br>2885	2926<br>2881	2928<br>2883	2928<br>2874	2928<br>2875	2927<br>2879
ν (C–O) from carbohydrate	1739	-	1739	1739	1739	1739
δ (O–H) from H_2O	1645	1646	1646	1645	1642	1645
δ (N–H) from amid II	1546	1546	1546	1546	1546	1546
δ (O–CH) and δ (C–C–H)	1449	1451	1448	1446	1449	1449
δ (O–H) in C–OH group + δ (C–H) in the alkenes	1417	1409	1417	1421	1419	1420
δ (–OH) in C–OH group	1363	1362	1365	1366	1366	1365
ν (C–H) in carbohydrates or/and ν(C–O) in carbohydrates	1258<br>1229	1252	1258<br>1230	1260<br>1229	1260<br>1228	1259<br>1229
ν (C–H) in carbohydrates or/and ν(C–O) in carbohydrates	1148	1145	1145	1148	1146	1147
ν (C–O) in C-O-C group	1101<br>1072	1101<br>1071	1098<br>1072	1102<br>1073	1101<br>1070	1100<br>1072

ν (C–O) in C–OH group or ν (C–C) in the carbohydrate structure	1017<br>990	1019<br>982	1015<br>990	1022<br>987	1023<br>987	1021<br>985
δ (C–H)	920	919	919	916	917	917
anomeric region of carbohydrates or δ (C–H)	897<br>864<br>843<br>817	893<br>865<br>817	896<br>864<br>844<br>816	894<br>868<br>818	895<br>867<br>844<br>817	896<br>866<br>855<br>818
